# Expression of TNF-Alpha-Dependent Apoptosis-Related Genes in the Peripheral Blood of Malagasy Subjects with Tuberculosis

**DOI:** 10.1371/journal.pone.0061154

**Published:** 2013-04-12

**Authors:** Niaina Rakotosamimanana, T. Mark Doherty, Lova H. Andriamihantasoa, Vincent Richard, Brigitte Gicquel, Jean-Louis Soares, Alimuddin Zumla, Voahangy Rasolofo Razanamparany

**Affiliations:** 1 Unité des Mycobactéries, Institut Pasteur de Madagascar, Antananarivo, Madagascar; 2 Staten Serum Institut, Copenhagen, Denmark; 3 Unité d'épidémiologie, Institut Pasteur de Madagascar, Antananarivo, Madagascar; 4 Unité de Génétique Mycobactérienne, Institut Pasteur, Paris, France; 5 Division of Infection and Immunity, University College London, London, United Kingdom; University of California, Riverside, United States of America

## Abstract

The majority of *Mycobacterium tuberculosis* (*Mtb*) infections remain asymptomatic with only up to 10% progressing to clinical tuberculosis. However, the constituents of the effective “protective immunity” against tuberculosis responsible for containing most infections remain unknown. Evaluating gene transcriptional profiles in tuberculosis clinical cohorts is one approach to understanding the spectrum of tuberculosis progression. It is clear that apoptosis plays a role in the control of tuberculosis but the utility of apoptosis-related genes as surrogate markers of protection against tuberculosis has not been well investigated. To characterize potential surrogate markers that could discriminate different phases of the clinical tuberculosis spectrum, we investigated gene expression of several TNF-alpha dependent apoptotic genes (TNFR1, TNFR2, FLICE, FLIPs) by real-time RT-PCR of peripheral blood cells from cohorts of individuals with active tuberculosis or potential exposure to tuberculosis.

Newly diagnosed tuberculosis patients (n = 23), their close household contacts (n = 80), and community controls (n = 46) were tested at intervals over a period of up to two years. Latent infection or previous *Mtb* contact was assessed by ELISPOT and TST and complete blood counts were performed during the follow up.

Results showed significant upregulation of FLIPs expression by infected individuals regardless of clinical status at entry to the study. A higher percentage of lymphocytes was found in the infected household contacts that remained healthy. In contrast, in individuals with active TB, a significant upregulation of TNFR2 expression, a significantly higher percentage of monocytes and a significantly decreased lymphocyte count were seen, compared to subjects that remained healthy. Moreover, the household contacts who subsequently developed signs of TB also had a significantly high number of monocytes.

These data suggest tuberculosis may be associated with decreased T-cell survival (perhaps due to apoptosis) while inhibition of apoptosis in monocytes could lead to a relative increase in these cells: a situation predicted to favour *Mtb*.

## Introduction

It has been estimated that a third of the world population is infected with bacteria from the *Mycobacterium tuberculosis* complex (MTC). These bacteria are the causal agents of tuberculosis (TB), a major cause of morbidity and mortality worldwide. Most infected individuals remain asymptomatic, but up to 10% can go on to develop active TB disease, becoming contagious during a period of months to decades after initial infection [1]. Current diagnostic tests for tuberculosis can detect previous exposure to members of the MTC. However, these tests cannot distinguish between previous infection and active disease, and this greatly hampers TB control programs. The development of effective diagnostic tests for TB and the identification of biomarkers of disease status are therefore urgently required. Furthermore, as the protective immune response to TB in humans has not been clearly defined, it is difficult to identify the infected individuals likely to develop active disease and requiring treatment. The vast numbers of individuals infected annually makes it impossible to consider treating all latent infections. The identification of risk factors for the development of active TB, and the monitoring of treatment success or of the protection provided by vaccines would all be vital steps towards containment of the TB epidemic [2], [3].

A strong cell-mediated immune (CMI) inflammatory response, involving tumor necrosis factor-alpha (TNF-α) and interferon-gamma (IFN-γ), is rapidly induced by infection with MTC and is required to protect the infected host against TB [4]. However, MTC can survive this inflammatory process and multiply within macrophages, by interfering with phagosome development, leading, in most cases, to a latent infection that may subsequently be reactivated, causing TB disease [3], [5]. Apoptosis seems to play an important role in the CMI response and TB control [6], [7]. The apoptosis of infected cells may limit bacterial growth by causing lysis of the bacteria within the apoptotic host cell, leading to the presentation of MTC antigens to T cells [8], [9]. It has also been suggested that the pathogen may use anti-apoptotic mechanisms to ensure its survival and growth within infected cells and to inhibit the development of T-cell immunity [10]. TNF-α appears to play a crucial role in reinforcing the host response to the pathogen [11] and TNF-α-dependent apoptosis seems to be a key element of immunity to TB. Various studies have suggested that molecules from the TNF-α family are involved in the apoptosis of macrophages or other cells infected with intracellular bacteria, including MTC [12]. For example, some virulent laboratory strains can induce the shedding of the TNF-α receptor (sTNFR2), which continues to bind its ligand, acting as a soluble antagonist of TNF-α preventing the lysis of infected host cells [13].

TNF-α acts through its membrane receptors, TNFR1 and TNFR2, which aggregate with other membrane and cytosolic proteins to form the “death-receptor complex” [14]. Signaling by these receptors initiates a cascade of reactions activating the proteins of the “death-signaling complex” (DISC), thereby initiating apoptosis [15] and limiting the replication of the intracellular bacteria [16]. Caspase-8 or FLICE is an essential pro-apoptotic component of the DISC, as is its antagonist, the FLICE-inhibitory protein or FLIP, which has a similar structure to FLICE but no catalytic activity and inhibits apoptosis. Recent observations have suggested that the DISC and certain “death-receptor domain” molecules are also involved in the activation and proliferation of T cells [17], [18]. The outcome of an MTC infection therefore probably depends on the balance between the various immune processes.

MTC may stimulate apoptotic death in a subset of T cells, by triggering the release of large amounts of TNF-α, while preserving their host cell by inhibiting the response to TNF-α and increasing the production of anti-apoptotic factors [19], [20], [21], [22], [23]. In this study, we tested this hypothesis in cohorts of TB patients, their recent household contacts and community controls from Madagascar, by using reverse transcriptase quantitative PCR (RT-qPCR) to assess the expression of the *TNFR1* and *TNFR2*, *FLICE* and *FLIPs* genes and evaluating cell counts.

## Materials and Methods

### Ethics statement

The participants were enrolled after they had received counseling and an explanation of the study. Only participants who gave written informed consent were included in this study. For minors and children, written informed consent was obtained from the next of kin.

The National Ethics Committee of the Ministry of Health of Madagascar approved the study (Authorization No. 038-SANPF/CAB, February 20^th^ 2004).

### Study site and subjects

Adult TB patients with a recent diagnosis based on a smear positive for acid-fast bacilli (AFB) (index cases [IC], over 15 years of age) were recruited at the principal anti-tuberculosis center in Antananarivo. Positivity was defined as two sputum samples classified positive by microscopy, with confirmation by culture on Lowenstein-Jensen medium. The household contacts (HC) of the included IC were visited at home by the study physicians and asked to participate in the study. They were included if they were at least one year old and had been living in the same house as the IC for at least six months. The subjects (or their legal guardians, for children) were informed about the study, their consent was then sought and they were interviewed and examined. Only subjects who agreed to undergo an HIV test, after counseling (where appropriate), and who had given informed consent were included in the study.

For every TB index case, two community controls (CC) were selected. These controls were healthy volunteers from the dispensary of the Pasteur Institute of Madagascar, matched for age and sex with two HC.

In total, we recruited 163 HIV-seronegative subjects: 25 IC, 88 HC and 50 CC. HC and CC had no TB symptoms and a chest X-ray on inclusion revealed no evidence of TB. Contacts were regularly monitored, at three month intervals, for up to two years after inclusion, to check for the development of TB symptoms.

For all subjects, epidemiological, clinical and bacteriological data were recorded prospectively on individual record forms. Blood samples were collected on inclusion in the study and at the end of eight months of anti-TB treatment for the IC. For HC and CC, blood samples were collected on inclusion and three months after inclusion.

### Blood tests and white blood cell count differences

Venous blood samples were collected into EDTA-coated Vacutainer tubes and stored at room temperature until analysis. White blood cell (WBC) count was determined with an automated ABX Pentra 120 Retic hematological analyzer (ABX, Montpellier, France). A biologist independently validated the assays.

Tuberculin skin test (TST) and ELISPOT assays were performed to check for previous exposure of the various subjects to MTC as described in a previous study [24]. Briefly, at the time of inclusion, physicians performed a standard Mantoux test and the results were read after 72 h. A cutaneous induration greater of 5 mm or more in diameter was considered to indicate a positive TST response and an induration of more than 14 mm in diameter was considered to indicate high reactivity. Peripheral blood mononuclear cells (PBMCs) were isolated from venous blood and stimulated with *M. tuberculosis* (*Mtb*) antigens for the quantification of IFN-γ production by ELISPOT. Briefly, PBMCs were isolated from heparinized whole blood by centrifugation on a Ficoll gradient. For each sample, we added 2×10^5^ PBMCs, in duplicate, to biotinylated anti-IFN-γ-coated 96-well plates (MAIP S 45; Millipore). PBMCs were stimulated with either PPD (*Mtb* purified protein derivative), or the 6 kDa early secretory antigenic target (ESAT-6) from *Mtb* and were incubated overnight, at 37°C, under an atmosphere containing 5% CO_2_, before staining by incubation with streptavidin peroxidase conjugate. The mean number of spot-forming cells (SFCs) of the negative control was subtracted from the number of SFCs per 2×10^5^ cells. The cutoff point for positivity was defined as the mean response of unstimulated wells for the whole cohort plus 1.64 standard deviations.

### RNA extraction and reverse transcription

Blood samples (2.5 ml) were collected in PAXgene blood tubes (PreAnalytix, Qiagen). Total RNA was extracted with the PAXgene RNA kit (PreAnalytix, Qiagen), according to the manufacturer's instructions, and RNA quality was assessed by checking for the presence of two rRNA bands on agarose electrophoresis gels and by quantification with a NanoDrop 1000 (Thermo Scientific). All samples were treated with RNase-Free DNAse (Qiagen) according to the manufacturer instructions before reverse transcription. We then generated cDNA from 300 ng of total RNA per sample, with the Omniscript RT kit (Qiagen) and oligo (dT) primers, according to the manufacturer's instructions. The cDNA aliquots were stored at −80°C until use.

### Quantification of the expression of apoptosis-associated genes by RT-qPCR

We assessed the expression of the *TNFR1*, *TNFR2*, *FLICE* and *FLIPs* genes, by carrying out RT-qPCR. The primers and dual-labeled probes (5′-FAM—TAMRA-3′) specific for each apoptotic marker studied were designed to span introns, to prevent amplification from genomic DNA (see list in Table 1). The QuantiTect Probe Master Mix kit (Qiagen) was used for real-time PCR, according to the manufacturer's instructions. Each reaction was performed in a volume of 25 µl, corresponding to 5 µl of cDNA and 20 µl of master mix. The gene encoding the human ribosomal protein HUPO was used as reference gene, to control for variation in the level of nucleic acid in the sample [25]. A Rotor-Gene (Corbett Research, Sydney, Australia) was used for real-time PCR. The PCR conditions were as follows: incubation for 15 minutes at 95°C, followed by 45 cycles of denaturation at 95°C for 10 seconds, annealing for 20 seconds and then extension at 72°C for 30 seconds. The fluorescence emitted was acquired at the end of every extension step. The reaction efficiency (range = 90–100%) was determined by serial dilution of a known quantity of plasmid clones for each gene, as previously described [26]. All reactions were performed in duplicate and the duplicate results had a standard deviation <0.4 of the mean. Controls, including tubes with no template for reverse transcription reactions, were included in each run. The numbers of copies of the mRNA for the various apoptosis-associated genes were then normalized to 10^5^ copies of the mRNA for the housekeeping gene encoding HUPO.

**Table 1:** Sequences of the primers and probes used to quantify gene expression by real-time PCR.

Gene	Nucleotide sequence	Product size (bp)	R^2^
*HuPO*	L: GCTTCCTGGAGGGTGTCC	105	0.99
	P: TGCCAGTGTCTGTCTGCAGATTGG		
	R: GGACTCGTTTGTACCCGTTG		
*TNFR1*	L: CGGTGGAAGTCCAAGCTCTA	114	0.98
	R: GGGACTGAAGCTTGGGTTT		
	P: CTGAAAAAGAGGGGGAGCTTGAAGGA		
*TNFR2*	L: ACCGTGTGTGACTCCTGTGA	101	0.99
	R: TCCACCTGGTCAGAGCTACA		
	P: ACTGGGTTCCCGAGTGCTTGAGCT		
*FLIP*	L: GTTCAAGGAGCAGGGACAAG	114	0.99
	R: ATCAGGACAATGGGCATAGG		
	P: TGGATTGCTGCTTGGAGAACATTCC		
*FLICE*	L: AAGTGCCCAAACTTCACAGC	102	0.98
	R: GGGGCTTGATCTCAAAATGA		
	P: ACTTGGATGCAGGGGCTTTGACCAC		

**L: Left primer (5′--------3′).**

**R: Right primer (5′--------3′).**

**P: Probe (5′ FAM--------TAMRA 3′).**

### Statistical analysis

The data obtained were analyzed by PCR with Rotor-Gene 6.0 software (build 23) from Corbett Research. Data were analyzed with Epi Info version 6.04dfr (CDC, USA and WHO, Switzerland) and groups were compared in nonparametric Kruskal-Wallis tests, with ANOVA used as a parametric tests, where appropriate (according to the variance comparison in Bartlett's chi-squared of variances homogeneity test). A *p* value<0.05 was considered statistically significant.

## Results

### Analysis of TB dynamics in a TB endemic country population

A full set of results was available for 149 (23 IC, 80 HC, 46 CC) of the 163 subjects who agreed to participate in the study. During follow-up, 10 HC from eight different families developed TB-like symptoms (symptomatic HC or sHC), although their AFB smears remained negative. These subjects were assumed to be possible cases of early-stage TB. The other 70 HC remained healthy (healthy HC or hHC) during the follow-up period.

The proportion of BCG-vaccinated subjects (ascertained on the basis of vaccination scars, vaccination declarations and a review of medical records) ranged from 80% to 91.3%, and no significant differences in this proportion were found between the four clinical groups (Table 2). Neonates are routinely vaccinated with BCG in Madagascar. The TST was negative for one third of the BCG-vaccinated subjects, and an induration >14 mm in diameter was observed in some individuals that had not been vaccinated (data not shown). No significant correlation was observed between the TST response and BCG vaccination status. No significant difference in TST response or PPD ELISPOT IFN-γ production was observed between the clinical groups (Table 2).

**Table 2:** Characteristics of the cohorts recruited for the study.

Cohort	IC	hHC	sHC	CC
**No. individuals**	23	70	10	46
**Mean age, years [range]**	32.48 [17–70]	21.94 [4–68]	18.1 [5–47]	22.35 [5–70]
**Sex M**	10	33	5	21
**F**	13	37	5	25
**TST at inclusion**				
**Negative**	2	17	2	24
**5–14 mm**	8	26	0	8
**≥15 mm**	7	27	8	14
**ND**	6	10	0	0
**BCG vaccination**				
**Yes**	20 (87%)	62 (88.5%)	8 (80%)	42 (91.3%)
**No**	2	3	1	1
**ND**	1	15	1	3
**PPD ELISPOT**				
**Negative (%)**	1 (4.3%)	11 (15.7%)	0	13 (28.3%)
**Positive (%)**	14 (60.9%)	37 (52.9%)	5 (50%)	24 (52.2%)
ND	8	22	5	9
**ESAT-6 ELISPOT**				
**Negative (%)**	5 (21.7%)	23 (32.9%)	3 (30%)	18 (39.1%)
**Positive (%)**	10 (43.5%)	25 (35.7%)	2 (20%)	19 (41.3%)
ND	8	22	5	9

### Differences in *TNFR2* and *FLIPs* expression in peripheral blood were associated with clinical status for TB

**Figure 1:**
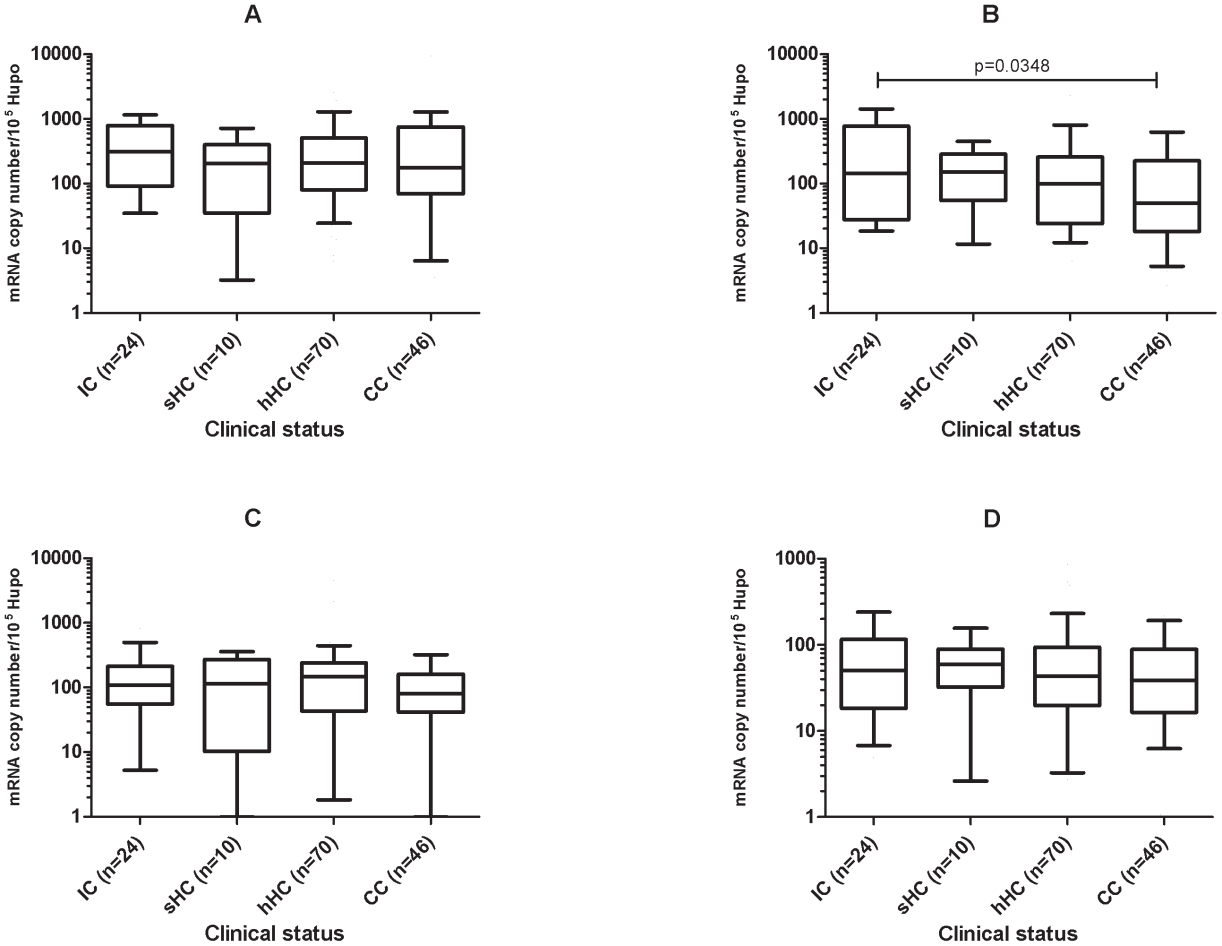
Expression of apoptotic genes in the blood between groups differing in clinical status for TB. (A) *TNFR1* expression, (B) *TNFR2* expression, (C) *FLIPs* expression, (D) *FLICE* expression. The data shown are the median and ranges of mRNA levels normalized and expressed as the number of copies per 10^5^ copies of mRNA for the housekeeping gene, *HuPO*. Mann-Whitney U tests were used for the pairwise comparison of groups. Significant differences in gene expression between clinical groups are indicated by a horizontal bar with the corresponding *p* value.

Total mRNA was extracted from blood samples collected on inclusion and at the various times during follow-up, as noted in the Materials and Methods. *TNFR1*, *TNFR2*, *FLIPs* and *FLICE* mRNAs were quantified with normalization with respect to 10^5^ copies of HUPO mRNA. Levels of TNFR2 mRNA were significantly higher in the IC than in the CC on inclusion (*p* = 0.03), and those of the HC were intermediate between these two groups (Figure 1B). The copy numbers of mRNA molecules for the other genes tested (*TNFR1*, *FLIPs* and *FLICE*) did not differ significantly between the clinical groups (*p* = NS, figure 1).

High levels of *TNFR2* expression were associated with TB disease. The levels of the four markers in IC at the end of anti-TB treatment were similar to those on inclusion in the study (*p*>0.05, data not shown).

**Figure 2:**
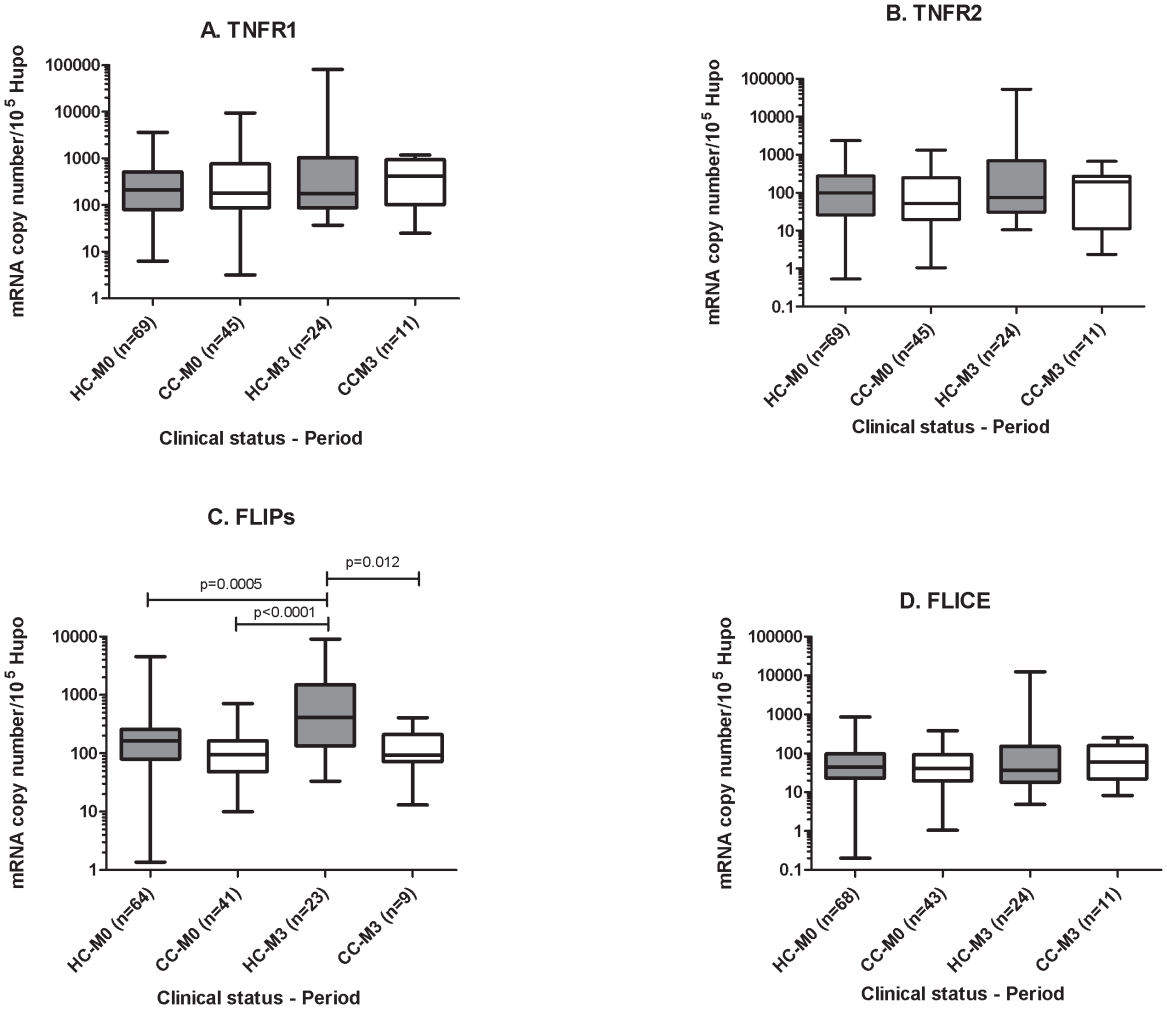
Comparison of peripheral blood gene expression in the contacts and controls after 3 months of follow-up. Expression on inclusion in the study (M0) and after 3 months of follow-up (M3) for (A) *TNFR1*, (B) *TNFR2*, (C) *FLIPs* and (D) FLICE. The data shown are the median and ranges for mRNA levels normalized and expressed as the number of copies per 10^5^ copies of the mRNA for the housekeeping gene, *HuPO*. Mann-Whitney U tests were used for the pairwise comparison of groups. Significant differences between the testing periods are shown. HC: household contact, CC: community control.

By contrast, *FLIPS* was significantly more strongly expressed after three months of follow-up than on inclusion in the HC (*p* = <0.01), whereas the level of expression of this marker remained unchanged in the matched community controls (figure 2C).

### *FLIPs* expression segregated according to TST and IFN-γ ELISPOT results

**Figure 3:**
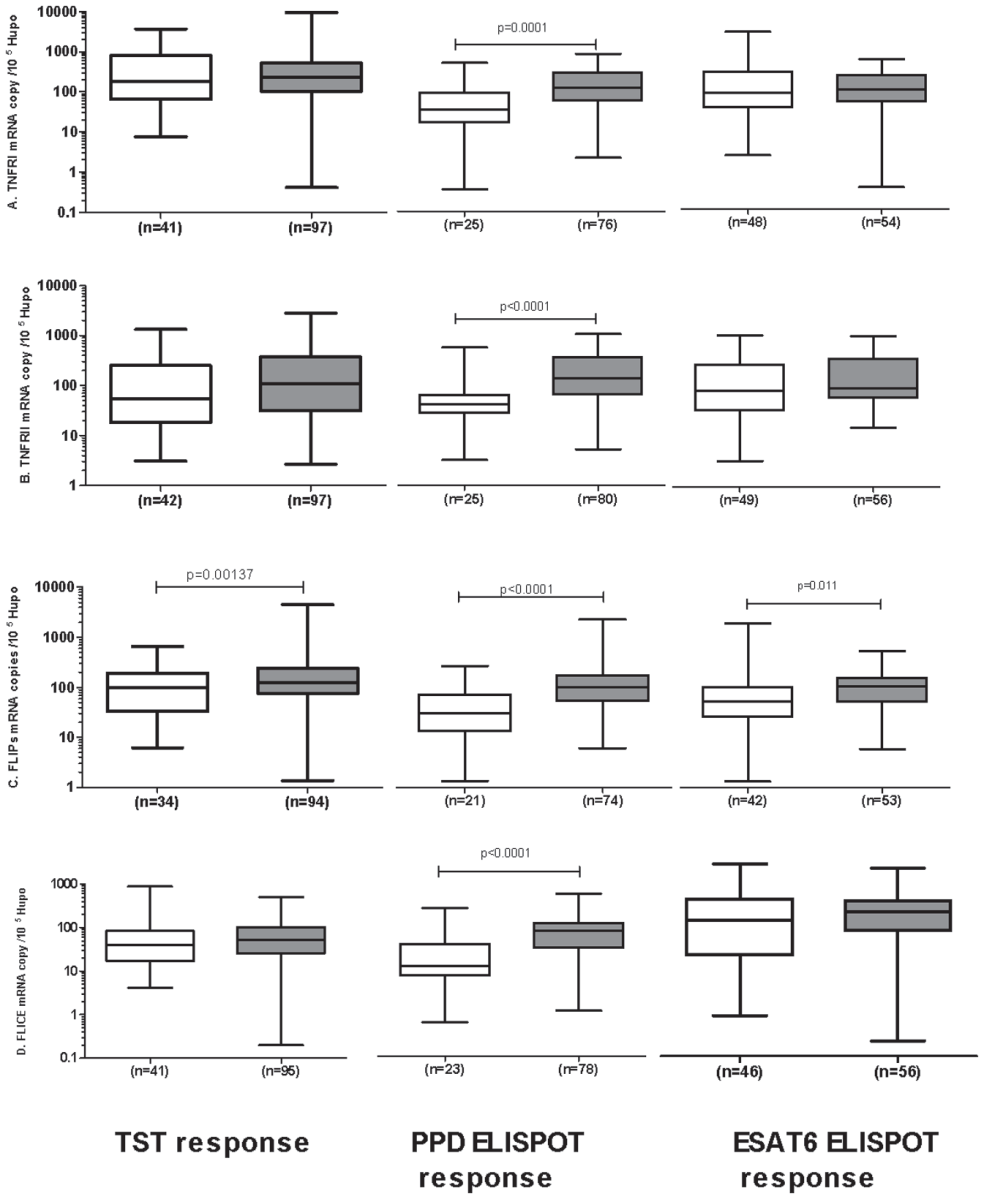
Peripheral blood gene expression as a function of TST, PPD-IFN-γ-ELISPOT and ESAT-6 ELISPOT responses. (A) *TNFR1*, (B) *TNFR2*, (C) *FLIPs* and (D) *FLICE*. The data shown are the median and ranges of mRNA levels normalized and expressed as the number of copies per 10^5^ copies of mRNA for the housekeeping gene, *HuPO*. The threshold for TST positivity was fixed at >5 mm. Neg, TST induration <5 mm, Pos, TST induration ≥5 mm in diameter. ELISPOT positivity was defined as described by Rakotosamimanana *et al.*, 2010. Individuals with a negative response are shown in white, those with a positive response in grey. Significant differences in gene expression between clinical groups are indicated.

**Figure 4:**
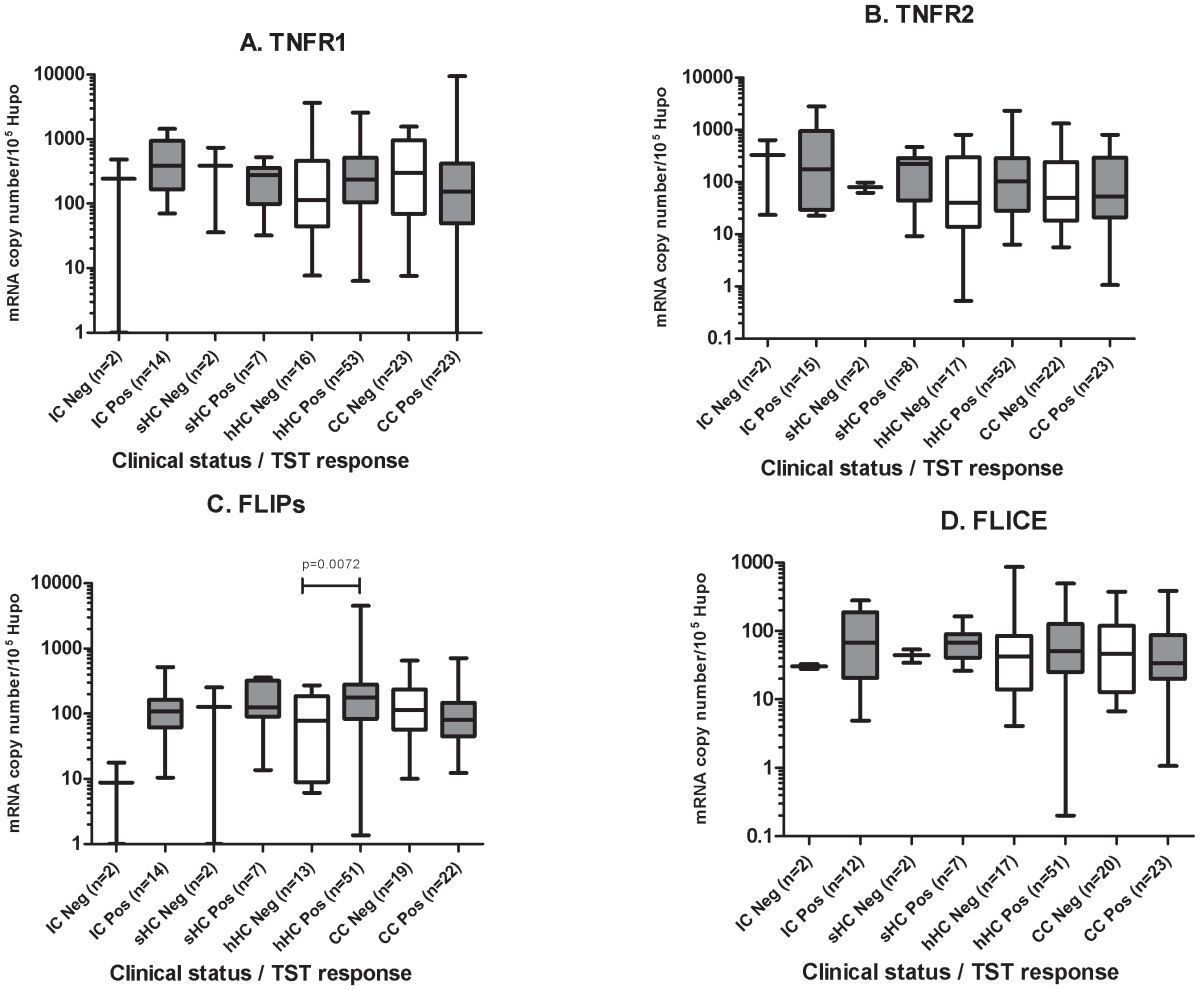
Blood expression of apoptotic genes as a function of TST response in the clinical groups. (A) *TNFR1*, (B) *TNFR2*, (C) *FLIPs*, (D) *FLICE*. TST positivity was defined as an induration >5 mm in diameter. Neg, TST induration <5 mm, Pos, TST induration ≥5 mm in diameter. The data shown are the median and ranges of mRNA levels normalized and expressed as a number of copies per 10^5^ copies of mRNA for the housekeeping gene, *HuPO*. Significant differences in gene expression between clinical groups are shown.

**Figure 5:**
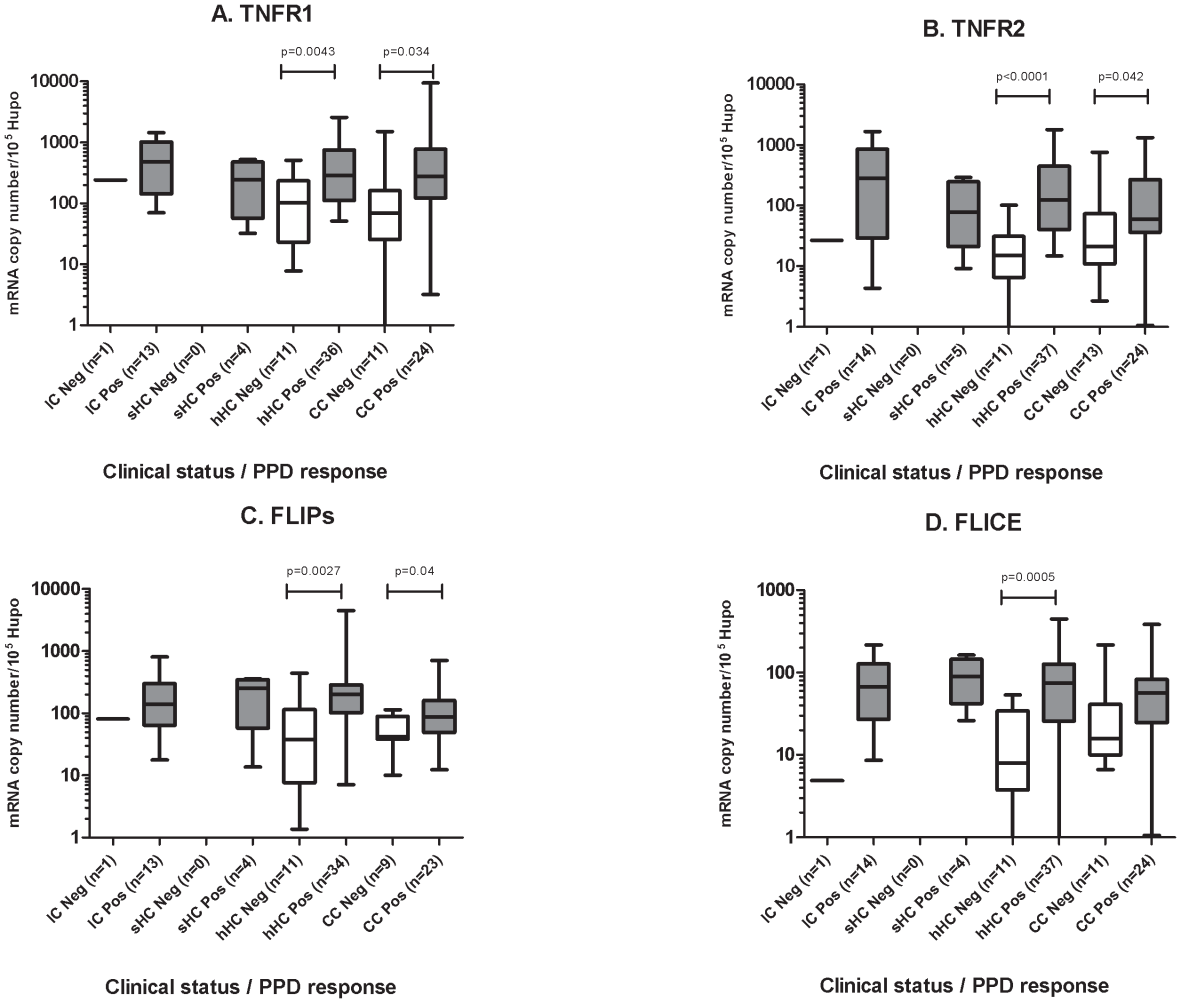
Peripheral blood gene expression as a function of PPD-IFN-γ-ELISPOT response in the clinical groups. (A) *TNFR1*, (B) *TNFR2*, (C) *FLIPs*, (D) *FLICE*. ELISPOT positivity was defined as described in the Materials and Methods. The data shown are the median and ranges of mRNA levels normalized and expressed as the number of copies per 10^5^ copies of mRNA for the housekeeping gene, *HuPO*. Significant differences in gene expression between clinical groups are indicated.

**Figure 6:**
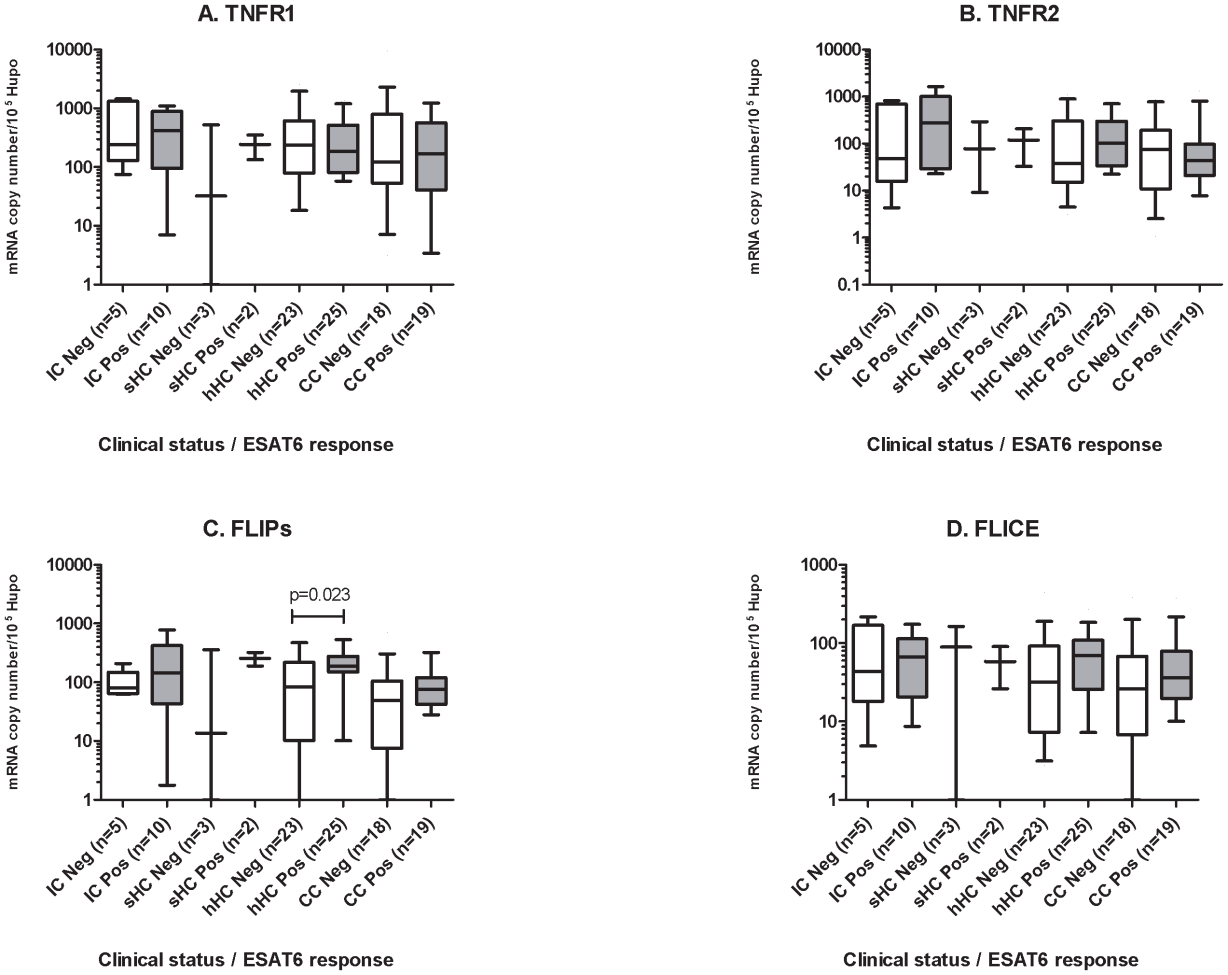
Peripheral blood gene expression as a function of ESAT-6 ELISPOT response in the clinical groups. (A) *TNFR1*, (B) *TNFR2*, (C) *FLIPs*, (D) *FLICE*. The data shown are the median and ranges of mRNA levels normalized and expressed as a number of copies per 10^5^ copies of mRNA for the housekeeping gene, *HuPO*. Significant differences in gene expression between clinical groups are indicated.

All groups, except the IC group who all have confirmed infection, had the potential to display heterogeneity in terms of *Mtb* exposure: not all contacts are necessarily infected (although we would expect most to be infected) and the CC group would be expected to include some with latent tuberculosis infection (LTBI). We therefore compared the expression of the target genes in TST-positive and TST-negative individuals. *FLIPs* expression was significantly stronger in the TST-positive (induration >5 mm) subjects than in the TST-negative subjects, in all clinical groups (figure 3C). A comparison of apoptotic gene expression segregated by PPD ELISPOT results showed that all the genes studied (*FLIPs*, *FLICE*, *TNFR1* and *TNFR2*) were more strongly expressed in PPD ELISPOT-positive individuals than in PPD ELISPOT-negative individuals (Figure 3). However, only *FLIPs* expression was significantly stronger in the ESAT-6 ELISPOT-positive individuals than in the ESAT-6 ELISPOT-negative individuals (*p*<0.05, figure 3), with no difference observed for the other genes studied. Furthermore, an analysis of each clinical group segregated by TST response showed that, within the hHC group, *FLIPs* expression was significantly stronger in individuals with a positive TST than in those with a negative TST (*p*≤0.01, figure 4) but no difference was seen for the other genes or clinical groups. All four genes were significantly more strongly expressed in the PPD ELISPOT-positive hHC and CC than in the non-responders (*p*<0.05, Figure 5). Nothing can be said about the IC group in this regard, since all but one were PPD, ELISPOT positive. Finally segregation of each clinical group by ESAT-6 responsiveness gave a result resembling the TST analysis. Within the hHC group, *FLIPs* expression was significantly stronger in those with a positive result for ESAT-6 ELISPOT than in hHC with a negative ESAT-6 ELISPOT result (*p* = 0.02, Figure 6).

This association of elevated expression of FLIPs with TST and ELISPOT positivity indicates it may be related to infection.

### Assessment of WBC composition in the different clinical groups

**Figure 7:**
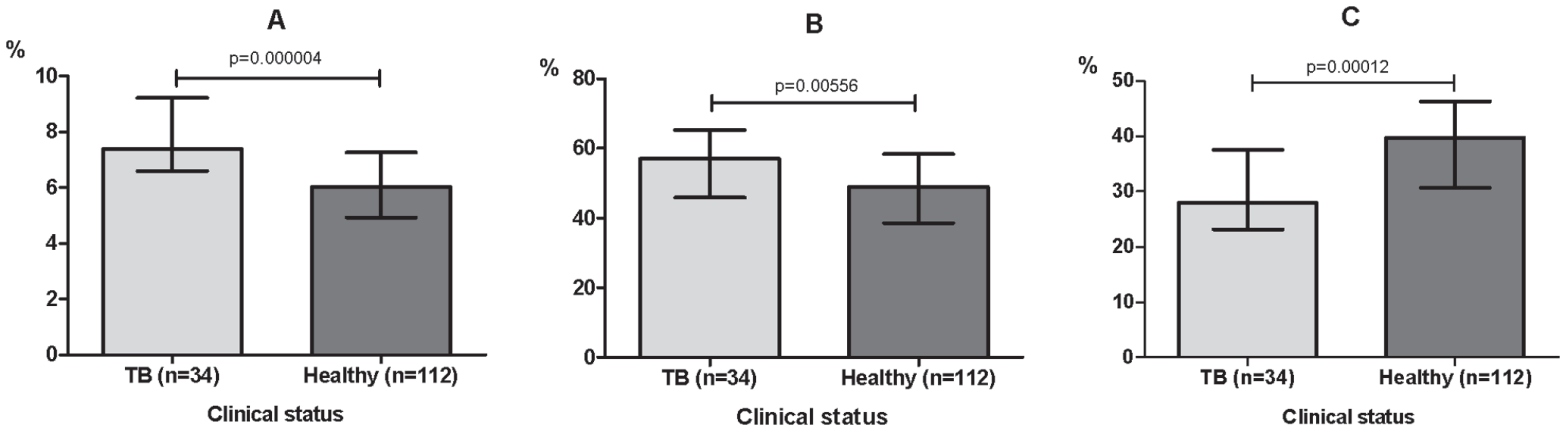
Peripheral blood cell distribution as a function of the presence or absence of TB symptoms. (A) Monocytes, (B) neutrophils and (C) lymphocytes. The data shown are the mean cell percentage+SD. Significant differences in gene expressions between clinical groups are indicated. TB, index cases and household contacts presenting TB symptoms.

We investigated the correlation between the expression of the apoptotic genes studied and differences in the composition of the WBC population, by analyzing the overall distribution of the WBC population (Table 3). Total WBC count was significantly higher in the hHC group than in the CC group (*p* = 0.02). Similarly, the TB patients (IC and sHC) had a significantly higher percentage of monocytes and neutrophils (*p*<0.05) but a lower percentage of lymphocytes, compared to the healthy subjects (hHC and CC) (Figure 7). Interestingly, this finding is compatible with recent data from 2 large cohort studies in India, using Multiplex ligation-dependent probe amplification, suggesting that it may be a generally applicable finding (Author's unpublished data). After treatment of TB patients, the neutrophil and the monocyte percentages decreased, while the lymphocyte percentage increased, erasing the difference between clinical groups (data not shown).

**Table 3:** Hematological characteristics.

Cohort	IC	hHC	sHC	CC	*p* value
**No. individuals**	24	69	8	46	
**RBC (×10^2^/l)**	4.792	5.101	4.875	4.935	0.130952
**Hemoglobin concentration (g/l)**	124.125	139.928	130.500	137.717	0.000427
**MCV (μ^3^)**	79.958	82.971	81.250	85.435	0.002287
**MCH (pg)**	26.750	27.957	27.375	28.478	0.009976
**MCHC (g/l)**	318.875	334.348	335.250	330.217	0.031949
**Leukocytes (×10^9^/l)**	6.250	6.870	6.375	5.826	0.130369
**Neutrophiles (%)**	56.958	48.768	50.375	48.591	0.028549
**Neutrophils (×10^9^/l)**	3.583	3.377	2.750	3.000	0.323279
**Lymphocytes (%)**	29.333	40.464	39.375	37.909	0.000726
**Lymphocytes (×10^9^/l)**	1.750	2.623	2.375	2.067	0.000157
**Monocytes (%)**	7.583	6.116	10.375	6.727	0.000079
**Monocytes (×10^9^/L)**	0.333	0.290	0.625	0.222	0.139802
**Platelets (×10^9^/l)**	397.958	291.072	371.750	287.000	0.000153

The mean values are given for each clinical group.

**MCHC:** mean corpuscular hemoglobin concentration; **MCV:** mean corpuscular volume; **MCH:** mean corpuscular hemoglobin (amount).

No significant correlation was observed between the expression levels of the four apoptotic genes studied and differences in WBC population distribution in the various clinical groups (IC with active TB, HC exposed to TB and CC; table 3).

### Apoptotic gene expression and WBC rates distinguish between healthy subjects, individuals with *Mtb* infection and individuals with active TB

As TB is endemic in Madagascar and the coverage rate is high for BCG vaccination, a weak TST response may not be specific for a *Mtb* infection. We thus defined infection as a strong TST response and assumed that healthy individuals with an induration in the TST<14 mm were potentially pre-sensitized to mycobacteria but not necessarily infected with *Mtb*, and further that healthy individuals, with a TST result<5 mm were most likely not infected. Those with a TST>14 were assumed to be infected with *Mtb*, even if asymptomatic

In infected healthy subjects, the number of copies of *FLIPs* mRNA in the hHC (177.78±219.9, n = 27) was greater than that in the CC group ((75.9±88.84, n = 15; *p*<0.01), while the levels of expression of the other genes studied did not differ between the two groups. The individuals with signs of TB disease (IC and sHC) also had higher levels of *TNFR2* mRNA in the peripheral blood than did healthy infected subjects with an induration in the TST>14 mm (*p* = 0.04; Table 4).

**Table 4:** Apoptotic gene expression in individuals with TST>14 mm.

Markers	TB (*n* = 15)	±SD	Healthy (*n* = 42)	±SD	*p-value*
*TNFR1*	276,1	381,3	178	406,5	0,9639
*TNFR2*	241,4	771	71,19	232,8	0.039
*FLIPs*	104,4	141,5	124,2	194,6	0,3794
*FLICE*	67,18	83,32	47,39	86,2	0,491

TB = active TB and symptomatic (IC+sHC). SD = standard deviation. The mean values are given for each clinical group.

**Figure 8:**
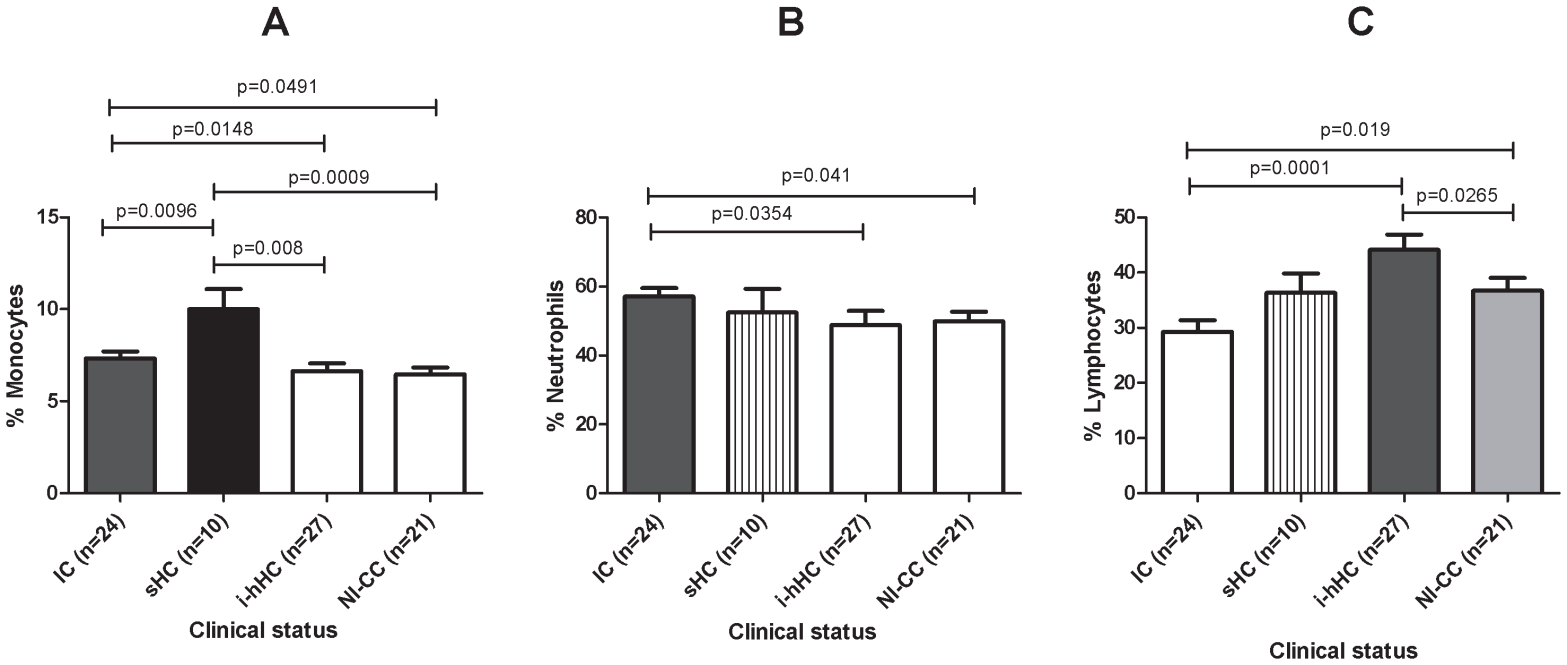
Peripheral blood cell proportions as a function of clinical status group. (A) Monocytes, (B) neutrophils and (C) lymphocytes. The data shown are the mean+SD of cell percentage. NI-CC = Non infected community control (CC with TST induration<5 mm), i-hHC = infected household contact (TST induration ≥14 mm), sHC = household contact that developed TB symptoms, IC = index TB case. Significant differences in gene expression between clinical groups are indicated.

The TB symptomatic individuals (IC and sHC) had significantly higher monocyte counts than the infected but healthy (i-hHC) or non infected individuals (NI-CC) ((*p*<0.05, figure 8A). The sHC had a percentage of monocytes, significantly higher than those of individuals with a different clinical status (figure 8A). The IC had a significantly higher proportion of neutrophils than the healthy individuals (i-hHC and NI-CC; figure 8B). Moreover, the healthy infected individuals (i-hHC) had significantly higher lymphocyte proportions than both the IC and the NI-CC (figure 8C).

**Figure 9:**
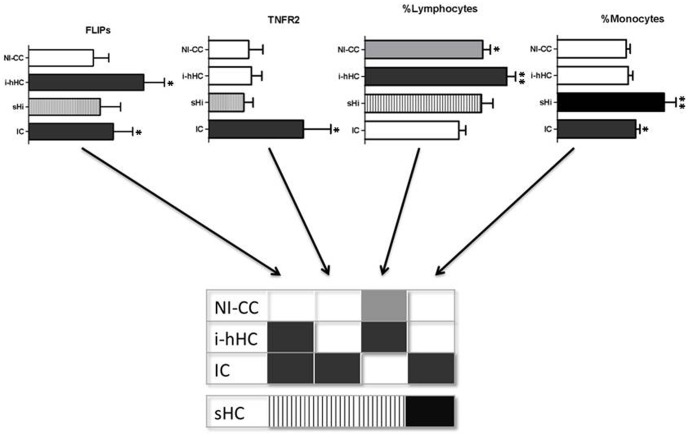
*FLIPs* and *TNFR2* expressions in combination with lymphocyte and monocyte proportions to characterize clinical status. NI-CC = Non-infected community control (CC with TST induration <5 mm), i-hHC = infected household contact (TST induration ≥14 mm), sHC = household contact developing TB symptoms, IC = index TB case. In dark gray: significant increase; in light gray: significant increase in community control; in black: significant increase in sHCs, hatched: non particular pattern of response identified. Significant differences are indicated with stars.

We tried to identify the characteristics distinguishing between those individuals who were able to control their infection and those who became ill (protected and non protected) by analysing the data on the factors which were different between the groups, namely *FLIPs* and *TNFR2* expression, and lymphocyte and monocyte counts. Combination of these four factors (Figure 9) gives a signature that could distinguish the IC group, infected hHC (i-hHC with TST>14 mm) and non infected CC (NI-CC with TST<5 mm). According to these parameters, high levels of *FLIPs* expression were associated with *Mtb* infection, but those who are able to maintain a higher ratio of lymphocytes and low monocyte levels are able to control this infection.

## Discussion

The progression of *Mtb* infection to active TB disease may take several years to decades, and infections may remain asymptomatic. Individuals with LTBI constitute a major reservoir of potential new active TB cases that could maintain the pandemic transmission of TB. This study was driven by the need for the characterization of surrogate markers of a protective immune response to *Mtb* infection or of progression to active TB disease.

Current diagnostic tests for TB cannot distinguish between active disease and latent *Mtb* infection. Some diagnostic tests are based on the cell-mediated immunity of the host against *Mtb* where a Th1 cytokine profile response has an important role, but this response is apparently unable to distinguish active disease from latent *Mtb* infection. TNF-α is an important part of this response, as indicated by the reactivation of disease by anti-TNF-α therapy [27]. We therefore looked at TNF-α-stimulated apoptotic genes since apoptosis of infected macrophages also plays an important role in controlling *Mtb* infection and regulating cell-mediated immunity to the pathogen [13], [15], [28], [29]. The expression of four genes associated with TNF-α-dependent apoptosis and downstream apoptotic effectors was quantified in the peripheral blood of patients with active TB, their household contacts and matched community controls.

As summarized in figure 9, the different clinical groups could be characterized on the basis of a combination of expression levels for *FLIPs* and *TNFR2* and the distributions of lymphocytes and monocytes in peripheral blood. Higher levels of expression of *FLIPs* in the peripheral blood seemed to be associated with *Mtb* infection, as *FLIPs* mRNA in the i-hHC and IC were significantly higher than those in the other groups. This higher level of *FLIPs* mRNA was also found in TST-positive and PPD or ESAT-6 ELISPOT-positive individuals. *FLIPs* gene expression was also significantly upregulated in the HC after three months of follow-up, as shown by comparisons with mRNA levels on inclusion in the study, while no such change was observed over the same period for the CC group, consistent with our hypothesis that elevated expression of *FLIPs* in this cohort reflects infection with *Mtb*.

However, not all *Mtb* infections lead to TB disease. The hHC identified as infected by TST and ELISPOT positivity who nonetheless remained healthy throughout the follow-up period had elevated expression of FLIPs but also an elevated percentage of peripheral blood of lymphocytes. This may reflect an active T cell response against *Mtb*. In the TB patients, elevated expression of *FLIPs* mRNA was also observed, but in this case, associated with a significant upregulation of *TNFR2* gene expression and an increased ratio of monocytes to lymphocytes. The fact that this ratio reverts to that seen in healthy individuals after successful treatment of TB is consistent with the idea that the T cell population may be inhibited in active TB. The contacts who developed TB-like symptoms in the course of follow-up (sHC) showed an intermediate pattern, with levels of FLIPs expression consistent with infection, and an elevated monocyte to lymphocyte ratio, but without significantly increased TNFR2 expression.

These results confirm and expand earlier work. In studies of TB patients, their household contacts and community controls from Ethiopia, Abebe and collaborators also observed a difference in apoptotic gene expression in the different clinical cohorts. This study suggested that monocytes from the Ethiopian TB patients were less sensitive to TNF-α-dependent apoptosis than the other cell lineages (notably T-cells), due to shedding of the TNFR2 receptor by monocytes [26]. These findings are consistent with the data reported here, and moreover provide a mechanism to explain these results.

The inhibition of TNF-α dependent apoptosis of infected macrophage has been suggested as a mechanism used by *Mtb* to preserve its intracellular niche and escape the host immune response [21] and it has been observed that virulent *Mtb* strains are able to directly inhibit TNF-α-dependent apoptosis of macrophages by activating the release of membrane-bound TNFR2 as the soluble form by infected host cells [13]. Similarly, observations from *Mtb*-induced apoptosis models suggested that FLIPs degradation was associated with TNF-induced apoptosis of *Mtb* infected macrophages. [15]. The higher level of FLIPs in *Mtb*-infected individuals in this study is concordant with the different *in vitro* observations and suggests that *Mtb*-induced increase of FLIPs may reflect an attempt by the pathogen to protect macrophages – the pathogen's preferred host cell – from TNF-α-dependent apoptosis. Whether this leads to latent infection or TB disease appears to correlate with the relative preservation or loss, respectively of lymphocytes in the peripheral blood of infected individuals, with a loss of T cell numbers correlating with the development of TB, as previously suggested [30], [31]. While most of these other studies were performed *in vitro* or in active TB patients, the current observations from the Malagasy cohort suggests that this mechanism is also active in infected household contacts and that early signs of monocyte/lymphocyte imbalance may identify those individuals who are failing to contain the infection.

Also supportive of our results, a recent microarray study on human TB has shown a significant decrease of lymphocytic cells and an increase of myeloid lineage transcripts in active TB patients, which was attributed to an expansion of inflammatory monocytes (CD14+CD16+) [31]. Further longitudinal studies to characterise monocytic subpopulations in TB contacts are therefore potentially very interesting.

The mechanism involved in the relative decrease in lymphocytes is as yet unclear. Observations from other studies in TB patients suggested a significant decrease in the number of certain *Mtb*-reactive T cells and a decreased production of IFN-γ was linked with activation of some apoptotic pathways [22], [23]. A Gambian study also found that a relative decrease in CD4 T cells in TB contacts was correlated with risk of subsequent TB, though the mechanism was not indicated [30]. The hypothesis that imbalances in regulation of apoptosis may lead to a loss of immune function and subsequent progress to TB is therefore an attractive one; however more work is required before we can say anything definitive about cause and effect. These results do however, highlight the importance of a better understanding of the role of apoptosis in the development of TB.

## Conclusions

In this study, we evaluated the utility of both gene expression and cell proportions, as combined markers for characterizing the protective response against TB in humans.

Changes in the expression of TNF-associated apoptotic genes seemed to be associated with changes in the distribution of immune cells in the peripheral blood of various clinical groups defined on the basis of TB status. An increase in *FLIPs* expression seemed to be associated with *Mtb* infection. In infected individuals who remained healthy, this *FLIPs* increase was associated with a higher ratio of lymphocytes to monocytes, while infected contacts who later developed TB-like symptoms showed the reverse pattern: a significant elevation of the ratio of monocytes to lymphocytes in the peripheral blood. TB index cases were also characterized by an elevated ratio of monocytes to lymphocytes and this reversed after successful treatment. Like infected contacts, TB patients had increased expression of *FLIPs*, when compared to healthy individuals but additionally displayed an increased level of expression of mRNA for TNFR2. Prior studies indicate that increased expression of the *TNFR2* gene by TB patients is associated with increased levels of serum soluble TNFR2 [26] which acts as a TNF-α antagonist, suggesting the same is likely true in this study.

While these data are the first to suggest that FLIPs might be a promising marker of *Mtb* infection, and that the combination of apoptotic genes and monocyte/lymphocyte markers may allow us to predict risk of progression from infection to full-blown TB, further studies are required to ascertain the usefulness of the observed parameters as surrogate markers of TB clinical status. Other factors influencing apoptosis and immune responses should be studied in a more integrative manner, with parallel studies of the genetics of human populations or *Mtb* strains, to improve our understanding of the disease and facilitate the development of new tools for combating tuberculosis.

## References

[1] Uriz J, Reparaz J, Castiello J, Sola J (2007) [Tuberculosis in patients with HIV infection]. An Sist Sanit Navar 30 Suppl 2: 131–142. 17898833

[2] Wallis RS, Pai M, Menzies D, Doherty TM, Walzl G, et al (2010) Biomarkers and diagnostics for tuberculosis: progress, needs, and translation into practice. Lancet 375: 1920–1937. 20488517 10.1016/S0140-6736(10)60359-5

[3] Russell DG (2001) Mycobacterium tuberculosis: here today, and here tomorrow. Nat Rev Mol Cell Biol 2: 569–577. 11483990 10.1038/35085034

[4] Kaufmann SH (2001) How can immunology contribute to the control of tuberculosis? Nat Rev Immunol 1: 20–30. 11905811 10.1038/35095558

[5] Hirsh AE, Tsolaki AG, DeRiemer K, Feldman MW, Small PM (2004) Stable association between strains of Mycobacterium tuberculosis and their human host populations. Proc Natl Acad Sci U S A 101: 4871–4876. 15041743 10.1073/pnas.0305627101 PMC387341

[6] Rook GA, Dheda K, Zumla A (2005) Do successful tuberculosis vaccines need to be immunoregulatory rather than merely Th1-boosting? Vaccine 23: 2115–2120. 15755581 10.1016/j.vaccine.2005.01.069

[7] Keane J, Balcewicz-Sablinska MK, Remold HG, Chupp GL, Meek BB, et al (1997) Infection by Mycobacterium tuberculosis promotes human alveolar macrophage apoptosis. Infect Immun 65: 298–304. 8975927 10.1128/iai.65.1.298-304.1997 PMC174591

[8] Perskvist N, Long M, Stendahl O, Zheng L (2002) Mycobacterium tuberculosis promotes apoptosis in human neutrophils by activating caspase-3 and altering expression of Bax/Bcl-xL via an oxygen-dependent pathway. J Immunol 168: 6358–6365. 12055253 10.4049/jimmunol.168.12.6358

[9] Schaible UE, Winau F, Sieling PA, Fischer K, Collins HL, et al (2003) Apoptosis facilitates antigen presentation to T lymphocytes through MHC-I and CD1 in tuberculosis. Nat Med 9: 1039–1046. 12872166 10.1038/nm906

[10] Abebe M, Kim L, Rook G, Aseffa A, Wassie L, et al (2011) Modulation of cell death by M. tuberculosis as a strategy for pathogen survival. Clin Dev Immunol 2011: 678570. 21253484 10.1155/2011/678570 PMC3022200

[11] Tsao TC, Hong J, Li LF, Hsieh MJ, Liao SK, et al (2000) Imbalances between tumor necrosis factor-alpha and its soluble receptor forms, and interleukin-1beta and interleukin-1 receptor antagonist in BAL fluid of cavitary pulmonary tuberculosis. Chest 117: 103–109. 10631206 10.1378/chest.117.1.103

[12] Hirsch CS, Johnson JL, Okwera A, Kanost RA, Wu M, et al (2005) Mechanisms of apoptosis of T-cells in human tuberculosis. J Clin Immunol 25: 353–364. 16133992 10.1007/s10875-005-4841-4

[13] Balcewicz-Sablinska MK, Keane J, Kornfeld H, Remold HG (1998) Pathogenic Mycobacterium tuberculosis evades apoptosis of host macrophages by release of TNF-R2, resulting in inactivation of TNF-alpha. J Immunol 161: 2636–2641. 9725266

[14] Wilson NS, Dixit V, Ashkenazi A (2009) Death receptor signal transducers: nodes of coordination in immune signaling networks. Nat Immunol 10: 348–355. 19295631 10.1038/ni.1714

[15] Kundu M, Pathak SK, Kumawat K, Basu S, Chatterjee G, et al (2009) A TNF- and c-Cbl-dependent FLIP(S)-degradation pathway and its function in Mycobacterium tuberculosis-induced macrophage apoptosis. Nat Immunol 10: 918–926. 19597496 10.1038/ni.1754

[16] Turner J, D'Souza CD, Pearl JE, Marietta P, Noel M, et al (2001) CD8- and CD95/95L-dependent mechanisms of resistance in mice with chronic pulmonary tuberculosis. Am J Respir Cell Mol Biol 24: 203–209. 11159055 10.1165/ajrcmb.24.2.4370

[17] Ch'en IL, Tsau JS, Molkentin JD, Komatsu M, Hedrick SM (2011) Mechanisms of necroptosis in T cells. J Exp Med 208: 633–641. 21402742 10.1084/jem.20110251 PMC3135356

[18] Oberst A, Dillon CP, Weinlich R, McCormick LL, Fitzgerald P, et al (2011) Catalytic activity of the caspase-8-FLIP(L) complex inhibits RIPK3-dependent necrosis. Nature 471: 363–367. 21368763 10.1038/nature09852 PMC3077893

[19] Oddo M, Renno T, Attinger A, Bakker T, MacDonald HR, et al (1998) Fas ligand-induced apoptosis of infected human macrophages reduces the viability of intracellular Mycobacterium tuberculosis. J Immunol 160: 5448–5454. 9605147

[20] Lopez M, Sly LM, Luu Y, Young D, Cooper H, et al (2003) The 19-kDa Mycobacterium tuberculosis protein induces macrophage apoptosis through Toll-like receptor-2. J Immunol 170: 2409–2416. 12594264 10.4049/jimmunol.170.5.2409

[21] Spira A, Carroll JD, Liu G, Aziz Z, Shah V, et al (2003) Apoptosis genes in human alveolar macrophages infected with virulent or attenuated Mycobacterium tuberculosis: a pivotal role for tumor necrosis factor. Am J Respir Cell Mol Biol 29: 545–551. 12748057 10.1165/rcmb.2002-0310OC

[22] Hirsch CS, Toossi Z, Vanham G, Johnson JL, Peters P, et al (1999) Apoptosis and T cell hyporesponsiveness in pulmonary tuberculosis. J Infect Dis 179: 945–953. 10068591 10.1086/314667

[23] Li B, Bassiri H, Rossman MD, Kramer P, Eyuboglu AF, et al (1998) Involvement of the Fas/Fas ligand pathway in activation-induced cell death of mycobacteria-reactive human gamma delta T cells: a mechanism for the loss of gamma delta T cells in patients with pulmonary tuberculosis. J Immunol 161: 1558–1567. 9686624

[24] Rakotosamimanana N, Raharimanga V, Andriamandimby SF, Soares JL, Doherty TM, et al (2010) Variation in gamma interferon responses to different infecting strains of Mycobacterium tuberculosis in acid-fast bacillus smear-positive patients and household contacts in Antananarivo, Madagascar. Clin Vaccine Immunol 17: 1094–1103. 20463103 10.1128/CVI.00049-10 PMC2897260

[25] Dheda K, Huggett JF, Bustin SA, Johnson MA, Rook G, et al (2004) Validation of housekeeping genes for normalizing RNA expression in real-time PCR. Biotechniques 37: 112–119, 112-114, 116, 118-119. 15283208 10.2144/04371RR03

[26] Abebe M, Doherty TM, Wassie L, Aseffa A, Bobosha K, et al (2010) Expression of apoptosis-related genes in an Ethiopian cohort study correlates with tuberculosis clinical status. Eur J Immunol 40: 291–301. 19877018 10.1002/eji.200939856

[27] Mohan AK, Cote TR, Block JA, Manadan AM, Siegel JN, et al (2004) Tuberculosis following the use of etanercept, a tumor necrosis factor inhibitor. Clin Infect Dis 39: 295–299. 15306993 10.1086/421494

[28] Hoft DF, Worku S, Kampmann B, Whalen CC, Ellner JJ, et al (2002) Investigation of the relationships between immune-mediated inhibition of mycobacterial growth and other potential surrogate markers of protective Mycobacterium tuberculosis immunity. J Infect Dis 186: 1448–1457. 12404160 10.1086/344359

[29] Nienhaus A, Schablon A, Diel R (2008) Interferon-gamma release assay for the diagnosis of latent TB infection–analysis of discordant results, when compared to the tuberculin skin test. PLoS One 3: e2665. 18628829 10.1371/journal.pone.0002665 PMC2441828

[30] Sutherland JS, Hill PC, Adetifa IM, de Jong BC, Donkor S, et al (2011) Identification of probable early-onset biomarkers for tuberculosis disease progression. PLoS One 6: e25230. 21966464 10.1371/journal.pone.0025230 PMC3179487

[31] Berry MP, Graham CM, McNab FW, Xu Z, Bloch SA, et al (2010) An interferon-inducible neutrophil-driven blood transcriptional signature in human tuberculosis. Nature 466: 973–977. 20725040 10.1038/nature09247 PMC3492754

